# International Society for Computational Biology Honours Gunnar von Heijne and Ziv Bar-Joseph with Top Bioinformatics/Computational Biology Awards for 2012

**DOI:** 10.1371/journal.pcbi.1002535

**Published:** 2012-05-31

**Authors:** Justin Mullins, BJ. Morrison McKay

**Affiliations:** 1 Freelance Science Writer, London, United Kingdom; 2 International Society for Computational Biology (ISCB), University of California San Diego, La Jolla, California, United States

## Introduction

Each year, the International Society for Computational Biology (ISCB; http://www.iscb.org) makes awards for exceptional achievement to two scientists. The ISCB Accomplishment by a Senior Scientist Award honours career achievement in recognition of distinguished contributions over many years in research, teaching, service, or any combination of the three. In 2012 this award is going to Gunnar von Heijne of the Stockholm University in Sweden. The Overton Prize recognizes a young scientist in the early to mid-stage of his or her career who has already achieved a significant and lasting impact in the field of computational biology. In 2012, the Overton Prize is being awarded to Ziv Bar-Joseph of Carnegie Mellon University in Pittsburgh, Pennsylvania, United States.

The recipients were chosen by the ISCB's awards committee chaired by Alfonso Valencia at the CNIO (Spanish National Cancer Research Centre) in Madrid. The winners will receive their awards at the ISCB's annual Intelligent Systems for Molecular Biology (ISMB) meeting, where they will deliver keynote talks. ISMB 2012 (http://www.iscb.org/ismb2012) marks the 20th anniversary of the conference, and will take place July 15–17 in Long Beach, California, United States.

## 2012 ISCB Accomplishment by a Senior Scientist Award: Gunnar von Heijne

Perhaps it all began with the French lessons. As a young PhD student in theoretical physics at the Royal Institute of Technology (KTH) in Stockholm, Gunnar von Heijne (Image 1) decided, on whim, to brush up on his rusty, schoolboy French. He took a few lessons and also subscribed to the French popular science magazine *La Recherche*.

**Figure:**
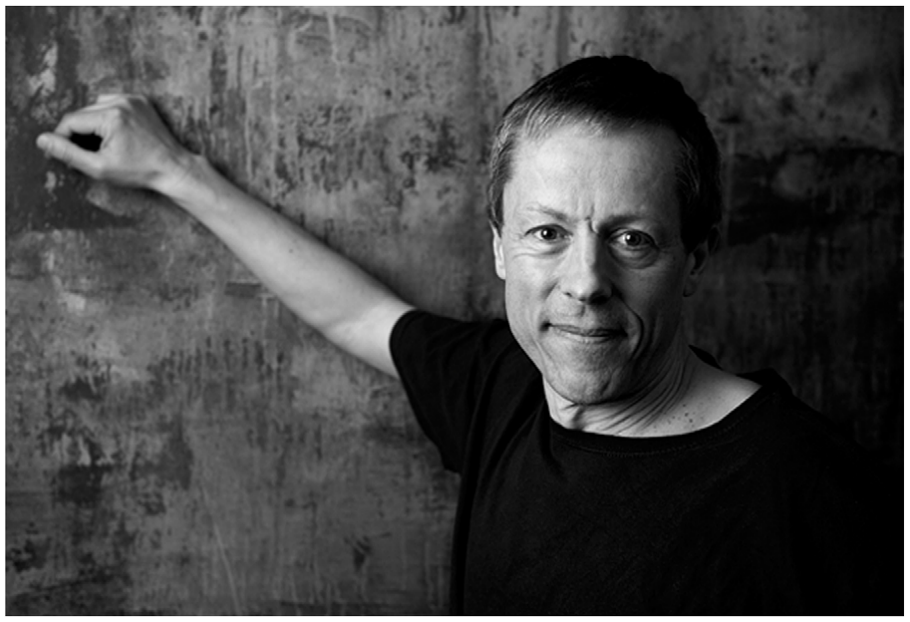
Image 1. Gunnar von Heijne of Stockholm University. Photo by Max Brouwers.

Flicking through its pages, he came across a short article on protein secretion and the signal hypothesis, the mechanism that describes the way secretory proteins cross a membrane.

At the time, the late-1970s, very little was known about this process, but some ideas were beginning to emerge. For example, it was thought that a so-called signal peptide—a short chain of amino acids—at the end of the protein carried the signal that determined how the proteins are transported out of the cell.

The article confused him, however. It showed a diagram of a hydrophobic signal peptide squeezing through the similarly hydrophobic membrane. “That didn't make sense to me. The hydrophobic peptide ought to become anchored in the membrane,” he says.

The puzzle piqued his interest. He solved it by calculating the energetics of a polypeptide chain passing through lipid bilayer, which he published in 1979. This work by a theoretician created ripples in a field dominated by experimentalists.

And so began the career for which he now receives the Accomplishment by a Senior Scientist Award from the International Society for Computational Biology (ISCB). “Gunnar is one of the big stars of our field,” says Burkhard Rost, president of the ISCB. “He is one of the few who completely change the field using computational methods.” Polypeptide energetics was only the start, however.

By the early 1980s, molecular biologists had begun to determine the sequence of amino acids in the signal peptides from different proteins. However, little had been done to study the properties of signal peptide sequences as a group.

von Heijne changed this. He began comparing the sequences, looking for recurring patterns that might help to identify them. “I looked at 20 to 30 signal peptides. Once you did that, some clear patterns emerged that had not been seen before,” he says.

He found that small, uncharged amino acids tended to occupy certain positions in signal peptide chains, the -3 and -1 positions. It is at this site that the signal peptide is later cleaved from the protein once it has passed through a biomembrane. This pattern has since become known as the (-3, -1)–rule.

“Nowadays you would say this was a very trivial bioinformatics study,” he says modestly. However, this was an important discovery and von Heijne's paper has since become one of the most highly cited in the field.

He then used the newly discovered patterns to make predictions about proteins. For example, it became possible to create an algorithm that would take a protein sequence and predict whether it had a signal peptide at the end.

Initially, that was not very useful. When molecular biologists sequenced a gene or messenger RNA, they generally knew what they were working on; whether it would have a signal peptide on the end or not.

But that changed when sequencing became faster and biologists started to sequence things they didn't know much about. “The algorithms have continually improved and are now extremely useful,” he says.

Secretory proteins have to move across a lipid bilayer through a molecular machine called a translocon. The signal peptide guides the ribosome that makes the protein, towards the translocon. This triggers the opening of this protein-conducting channel through the membrane.

But other types of protein only make the journey partway, becoming embedded half in and half out of the membrane. These so-called membrane proteins use the same translocon machinery as the secretory proteins. “So it was a natural step to start looking at these membrane proteins next,” says von Heijne.

The part of the protein that ends up in the membrane is very different to the parts outside exposed to water. This transmembrane section must be much more hydrophobic. So the trick to predicting which parts of a protein become embedded in the membrane is to look for the segments that are most hydrophobic.

Once you know the transmembrane segments, an interesting problem is to determine how the protein becomes woven into the membrane. For example, if it has four hydrophobic sections, there are two ways in which it can be arranged in the membrane: with the termini pointing either in or out. But which orientation should the protein take?

“We discovered a very simple principle that determines this,” he says. The regions that connect the transmembrane segments contain positively charged amino acids, which give them an electric potential. The simple principle is that the segments with the greatest number of positive charges end up inside the membrane, an idea that has since become known as the “positive inside rule”.

“This is very important work and provides some of the best data on membrane proteins,” says Valencia, chair of the ISCB awards committee.

In the late 1980s, von Heijne began to realise that he could gain significant insight into these and other problems by doing experiments rather than just theory work. So he set up his own lab. “I trained as a chemist so I wasn't a complete novice in a wet lab,” he says.

This first idea was to see whether it was possible to make proteins that inserted “upside-down” into the membrane. He could show that by changing the location of the positively charged amino acids in a protein, it is possible to make it take up the opposite orientation.

This link between his theoretical and practical work has been important for him. Bioinformatics studies often throw up patterns that may or may not have biological relevance. “The only way to determine whether they are important is to do the experiments,” he says.

“It's hard to overstate the significance of von Heijne's work. Membranes and transmembrane proteins are the gates and gatekeepers to our cells; they determine what gets in and what stays out,” explains Rost. “That's why around two-thirds of drugs target membrane proteins.”

Understanding the structure of transmembrane proteins provides crucial insight into how cells work and is also useful for future drug development. “That's why the methods developed by Gunnar are so important,” says Rost.

To continue his work, von Heijne set up the Stockholm Bioinformatics Centre at the beginning of the millennium. And today, von Heijne runs the Centre for Biomembrane Research in Stockholm, where he has brought together computational, modelling, and experimental groups. Few places can boast the same breadth of experience under one roof.

Throughout this time, von Heijne has maintained an impressive work–life balance as a scientist, a husband, and a father. He says that's been possible, at least in part, because he was working in a new field with few competitors. “I never felt stressed that we'd be scooped. I work hard but not crazily.”

Others clearly admire his positive approach, which he combines with a relaxed attitude. “He also looks ten years younger than he has any right to!” says one envious colleague.

For a while in the 1980s, he spent half his time working as a science journalist for the Swedish National Radio. “You decide on Monday what you broadcast on Friday so there is immediate feedback, which has a good pulse to it,” he says.

But for von Heijne, doing science is more satisfying than reporting it. “Radio stories have a short half life; they're on air, then they're gone,” he says. “The rewards in science are greater and longer lasting.” It's surprising how far schoolboy French can take you.

## 2012 ISCB Overton Prize: Ziv Bar-Joseph

Ziv Bar-Joseph (Image 2) loves to run. He rises early and hits the streets and trails around Pittsburgh where he lives, often in training for a long-distance race. This dedication has paid off. He has the enviable distinction of having run a sub-three hour marathon, a feat achieved by few amateurs. “Running is very important to me,” he says.

**Figure:**
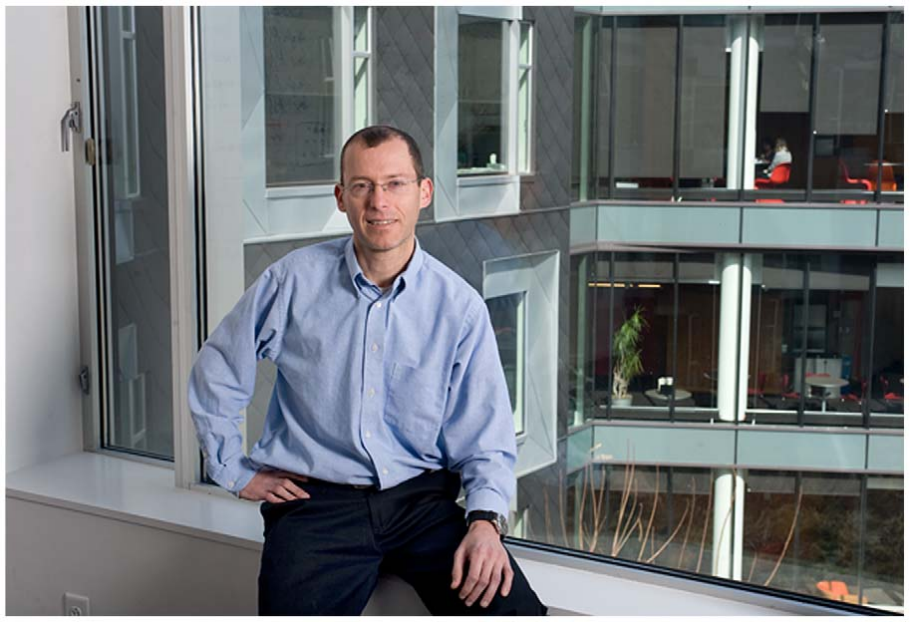
Image 2. Ziv Bar-Joseph of Carnegie Mellon University. Photo courtesy of Carnegie Mellon University.

But it is not just in his running that Bar-Joseph shows a willingness to go the distance. As a computer scientist and computational biologist at Carnegie Mellon University in Pittsburgh, Bar-Joseph shows a similar dedication as head of the Systems Biology Group at the School of Computer Science. “We have all been impressed by the quality of his scientific contribution and the novelty of the approaches he has developed,” says Valencia.

Bar-Joseph gained a PhD in computer science from the Massachusetts Institute of Technology between 1999 and 2003. That time turned out to be hugely significant, not least because computational biology was undergoing a revolution. “For the first time, we were getting sequences for large species. First, the fly, then humans. It was very inspiring,” he says.

Initially, Bar-Joseph knew little about computational biology but took a class to better understand the significance of these advances and the problems they posed. “It seemed to me that these types of problems were well-suited for the machine learning tools I had experience with,” he says.

One of the key problems was how to compare sequences either within species or between them. Various researchers had developed methods to do this using a branch of computer science called combinatorics, which essentially counts the number of similar patterns.

But while this works well when comparing two sequences, it's not so good for comparing seven or eight sequences. It doesn't scale. Consequently, researchers began to experiment with probabilistic approaches that focus on the statistical properties of the patterns. In particular, computational biologists had significant successes with a statistical approach called a hidden Markov model. That attracted Bar-Joseph who had studied this model.

He also recognised that other earlier studies, attempting to reconstruct networks in cells, were significantly limited: the data was a snapshot of a complex dynamic system but they treated it as if it were static.

Clearly, biological systems change. “One thing I've been involved in is introducing dynamics into the algorithms so that they can cope with the way things change in time. That requires different tools,” he says.

The approach has paid off when it comes to understanding regulatory networks and explaining how proteins control each other. For example, yeast has about 6,000 proteins. Of these, some 250 are control proteins and each of these, on average, controls 100 or so other proteins. However, each control protein is itself controlled by a handful of other proteins.

Understanding a system like this is a tricky business. The static data can tell you what proteins control other proteins, but that doesn't tell you when and under what conditions because that requires more experiments.

Other types of data are more temporal and can reveal how protein levels change over time. “The question we asked was whether we can use this temporal data to try and recover the underlying network dynamics,” he says. “We came up with methods to integrate these datasets in order to reconstruct the set of events over time and these have since been used in various other systems too.”

Bar-Joseph has learnt to work closely with biologists who test the results. “If the algorithm predicts that ‘a’ controls ‘b’, for example, you can do the experiment to test whether that's true.” That's important because the patterns that the algorithms reveal must be biologically relevant.

To better understand the challenges that experimentalists face, Bar-Joseph spent a sabbatical working in a wet lab doing exactly this kind of work. That taught him some valuable lessons. For example, wet lab work is not just a question of validating the model. “The results from the lab feed back into the model and enhance it. It's a two-way street,” he says.

Others have been impressed with Bar-Joseph's approach to experimental work. “Ziv is an example of somebody coming from the theoretical side of things and completely embracing the experimental approach,” says Rost, president of the ISCB. “It's stunning how he is able to handle such a diverse set of technical methods.”

This process of feedback from biology to computer science has become an important theme in Bar-Joseph's work. One of his recent successes is in explaining the way fruit flies develop bristles on their foreheads. These bristles are like aircraft sensors, measuring temperature, wind speed, and so on. To work well, they need to be spaced in a very precise way.

The bristles grow from cells but clearly only a small subset of cells. The cells do not know how many neighbours they have or the local density of bristles nearby. So what determines which cells grow into bristles and the spacing between them?

Bar-Joseph quickly realised that this was similar to a problem that computer scientists have wrestled with for 30 years. This is the problem of determining the subset of computers in a network that control all the others. When each computer in the network is connected to one computer in this subset (but no two in the subset are connected to each other), this subset is called maximally independent.

Finding maximally independent sets is hard, particularly in large distributed networks. Computer scientists do it by assuming that every computer knows who all its neighbours are.

Bar-Joseph realised that the fruit fly cells that eventually become bristles form a maximally independent set—they are connected to all other cells but not to each other. However, they do not know who their neighbours are and so must solve this problem in a different way. His breakthrough was to work out how they did it and develop an algorithm that does the same thing while assuming no knowledge of the neighbours. “It takes a bit longer but that's the trade-off,” he says.

This may have important applications for wireless sensor networks that researchers are using to monitor everything from ocean conditions to volcanic eruptions. “We only published at the beginning of 2011 so we don't know if it will penetrate the commercial world,” he says.

Valencia is also impressed by Bar-Joseph's broader contribution to the computational biology community. “He is a member of the editorial board for the journal *Bionformatics*, so clearly his contributions go beyond this theoretical and experimental work,” says Valencia. “That's very good for a young scientist.”

The future holds many promising problems for Bar-Joseph too. He is particularly interested in studying how pathogens interact with cells, how the proteins from flu viruses interact with cell proteins, for example. “If we can reconstruct the networks of interactions then we might be able to determine intervention points that will guide us to therapeutics,” he says.

He also wants to study the interaction networks in different species. Many of the genes in humans and mouse are similar, but drugs that work well in mouse often don't work in humans because the pathways, levels, and interactions are different. “We want to get more insight into this,” he says.

That's clearly a long game. These are problems that will require dedication, talent, and endurance to solve. Exactly the kind of qualities you might find in a marathon runner.

## Additional Information

The full conference agenda and registration information for ISMB 2012, including details on when these ISCB award winners plus four other distinguished keynote lecturers will be speaking, can be found on the conference web site at http://www.iscb.org/ismb2012. The conference will also feature parallel tracks for proceedings of original research papers, highlights of recently published papers, special sessions on emerging topics, late-breaking research of peer-reviewed abstract submissions, technology demonstrations, and workshops presented by academic researchers, funding agency representatives, and commercial vendors. The conference also offers a commercial and non-profit vendor exhibition.

For a review of past ISCB award winners, please see http://www.iscb.org/iscb-awards.

